# Benzylamides and piperazinoarylamides of ibuprofen as fatty acid amide hydrolase inhibitors

**DOI:** 10.1080/14756366.2018.1532418

**Published:** 2019-01-27

**Authors:** Alessandro Deplano, Mariateresa Cipriano, Federica Moraca, Ettore Novellino, Bruno Catalanotti, Christopher J. Fowler, Valentina Onnis

**Affiliations:** a Department of Life and Environmental Sciences – Unit of Pharmaceutical, Pharmacological and Nutraceutical Sciences, University of Cagliari, Cagliari, Italy;; b Department of Pharmacology and Clinical Neuroscience, Umeå University, Umeå, Sweden;; c Department of Chemical Sciences, University of Napoli Federico II, Napoli, Italy;; d Department of Pharmacy, University of Napoli Federico II, Napoli, Italy

**Keywords:** Ibuprofen amides, FAAH inhibition, fatty acid amide hydrolase, endocannabinoids, induced fit docking

## Abstract

Fatty Acid Amide Hydrolase (FAAH) is a serine hydrolase that plays a key role in controlling endogenous levels of endocannabinoids. FAAH inhibition is considered a powerful approach to enhance the endocannabinoid signalling, and therefore it has been largely studied as a potential target for the treatment of neurological disorders such as anxiety or depression, or of inflammatory processes. We present two novel series of amide derivatives of ibuprofen designed as analogues of our reference FAAH inhibitor Ibu-AM5 to further explore its structure-activity relationships. In the new amides, the 2-methylpyridine moiety of Ibu-AM5 was substituted by benzylamino and piperazinoaryl moieties. The obtained benzylamides and piperazinoarylamides showed FAAH inhibition ranging from the low to high micromolar potency. The binding of the new amides in the active site of FAAH, estimated using the induced fit protocol, indicated arylpiperazinoamides binding the ACB channel and the cytosolic port, and benzylamides binding the ACB channel.

## Introduction

**Figure 1.**
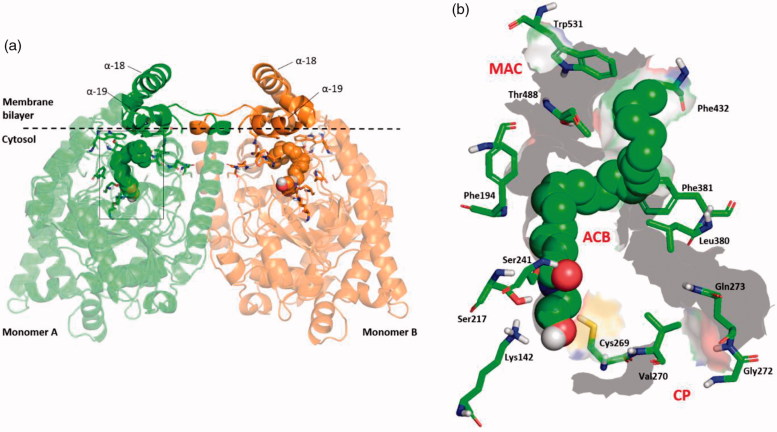
(a) 3D structure of the homo-dimer rat FAAH (*r*FAAH) model complexed with Anandamide (AEA). Monomer a and b are shown as green and orange cartoon, respectively. The membrane bilayer is indicated as dashed black line. (b) Details of the rFAAH binding cavity and channels. Key aminoacids of the binding cavity are highlighted as green sticks: Ser217:Ser241:Lys142 (catalytic triad), membrane access channel (MAC), the cytosolic port (CP) and the acyl-chain binding pocket (ACB).

N-acylethanolamines (NAE) are endogenous lipid ligands that regulate numerous physiological functions in the body due to activation of cannabinoid receptors, peroxisome proliferator-activated receptor-alpha (PPAR-α), and other targets^1^. Arachidonoylethanolamide (anandamide, AEA), palmitoylethanolamide, oleoylethanolamide, stearoylethanolamide and linoleoylethanolamide are the principal *N*-acylethanolamines. Fatty acid amide hydrolase (FAAH) is a serine hydrolase enzyme largely responsible for the hydrolytic degradation of *N*-acylethanolamines. The FAAH catalytic mechanism exploits an unusual catalytic triad, Ser-Ser-Lys, in which the basic Lys142 activates the nucleophilic Ser241, involving the Ser217 as a “proton shuttle”^2^. Structurally, FAAH is a homodimer enzyme bound to the membrane^3^ (Figure 1(a)). Its binding cavity is characterised by a series of separate channels that are crucial for its biological function: (i) the membrane access channel (MAC) that connects the membrane-bound region with the enzyme active site; (ii) the acyl-chain binding channel (ACB) including the catalytic triad and residues involved in the substrate binding; (iii) the cytosolic port (CP), which represents a way out for the hydrophilic product of the substrates hydrolysation^4^ (Figure 1(b)).

A number of different classes of FAAH inhibitors have been described in the literature, including carbamate derivatives, α-ketoheterocycles, piperazinyl, and piperidinyl ureas and boronic acids^5^. Inhibition of FAAH increases NAE levels in the brain and other tissues, but does not produce the sorts of behaviours seen with Δ-tetrahydrocannabinol, the main psychoactive ingredient of cannabis^6^^,^^7^ thereby making the enzyme a potentially exciting target for drug development. In humans, most FAAH inhibitors are well-tolerated^8–10^, the exception being BIA10-2474 which produced its toxic effects by presumed off-target effects^11^^,^^12^. In animal models, FAAH inhibition produces potentially beneficial effects in a variety of animal models of pain, but this has not been translated into the clinic^10^^,^^13^^,^^14^. However, other indications remain of great interest, not least in the field of anxiety/depression^15^^,^^16^ and intestinal inflammation^17^^,^^18^.

In 1997, it was reported that the non-steroidal anti-inflammatory drug ibuprofen inhibited FAAH^19^. Although the potency was modest, the IC_50_ concentration was in the range that could be achieved in humans. The ability of ibuprofen to inhibit FAAH is shared by other profens such as flurbiprofen^20^ and carprofen^21^. In previous studies, our research group has reported the FAAH inhibitory activity of profen amides and showed that the amide of Ibuprofen with 2-amino-3-methylpyridine (**Ibu-AM5**) (Table 1) was two to three orders of magnitude more potent than ibuprofen itself as a reversible inhibitor of FAAH^22^^,^^23^. The compound has a much lower ulcerogenic potency than ibuprofen^24^. In other studies, we have explored the SAR of **Ibu-AM5** analogues by modifying the 2-aminopyridine moiety^25^ and the isobutyl moiety^26^. Here, we present the synthesis, docking studies, and pharmacological characterisation of two new series of Ibuprofen derivatives, the benzylamides, and the piperazinoamides.

**Table 1.** Maximum percentage and IC50 values for inhibition of rat brain AEA hydrolysis by compounds **3–16**.

											
											
											

*Values with ethanol as solvent, taken from^25^. For the test compounds, the solvent was ethanol except when indicated with †, where DMSO was used. §The inhibition data was better fitted by a curve with a residual activity rather than a curve assuming 100% inhibition. The maximal inhibition is indicated (when it was greater than 50%), and the pI50 and IC50 values refer to the inhibitable portion of the curve. The inability of the compounds to produce a maximal inhibition was not investigated further. #Values for URB-597, as reference, with a preincubation time of 60 min, are taken from^35^.

## Experimental

### Materials

Anandamide [ethanolamine-1-^3^H] (specific activity 2.22 TBq mmol-1) was purchased from American Radiolabeled Chemicals, Inc (St. Louis, MO). All commercially available solvents and reagents were used without further purification and were purchased from Sigma-Aldrich (Milan, Italy).

### Chemistry

NMR spectra were recorded on an Inova 500 spectrometer (Varian, Palo Alto, CA). The chemical shifts (δ) are reported in part per million downfield from tetramethylsilane (TMS), which was used as internal standard, and the spectra were recorded in hexadeuteriodimethylsulphoxide (DMSO-d_6_). Infra-red spectra were recorded on a Vector 22 spectrometer (Bruker, Bremen, Germany) in Nujol mulls. The main bands are given in cm^−1^. Positive-ion electrospray ionisation (ESI) mass spectra were recorded on a double-focusing MAT 95 instrument (Finnigan, Waltham, MA) with BE geometry. Melting points (mp) were determined on a SMP1 Melting Point apparatus (Stuart Scientific, Stone, UK) and are uncorrected. All products reported showed ^1^H NMR spectra in agreement with the assigned structures. The purity of the tested compounds was determined by combustion elemental analyses conducted by the Microanalytical Laboratory of the Chemistry Department of the University of Ferrara with a MT-5 CHN recorder elemental analyser (Yanagimoto, Kyoto, Japan) and the values found were within 0.4% of theoretical values.

### General procedure for the synthesis of benzylamide derivatives 3–16

A solution of ibuprofen **1** (0.21 g, 1 mmol), 1–(3-dimethylaminopropyl)-3-ethylcarbodiimide hydrochloride (EDC) (0.19 g, 1.1 mmol) and hydroxybenzotriazole (HOBt) (0.13 g, 1 mmol) in anhydrous acetonitrile (MeCN) (10 ml) was stirred at r.t. for 30 min. Then the appropriate substituted benzylamine **2** (1 mmol) was added. The mixture was then stirred for 24 h at r.t. After the solvent was removed under vacuum, the residue was dissolved in ethyl acetate (AcOEt) (20 ml) and washed sequentially with brine (2 × 5 ml), 10% citric acid (2 × 5 ml), NaHCO_3_ 10% aqueous solution (2 × 5 ml) and water (2 × 5 ml). The organic layer was dried over anhydrous Na_2_SO_4_ and evaporated under vacuum. The obtained residue was tritured with iPr_2_O; the precipitate was then filtrated to obtain the compounds **3–16.**

### N-Benzyl-2–(4-isobutylphenyl)propanamide (3)

Obtained following the general procedure by the condensation between **1** and benzylamine. Yield 78%. m.p. 60–62 °C. ^1^H NMR (DMSO-d_6_) δ 0.85 (d, *J* = 6.5 Hz, 6H, CH_3_), 1.34 (d, *J* = 7.0 Hz, 3H, CH_3_), 1.81 (hept, *J* = 6.5–7.0 Hz, 1H, CH), 2.41 (d, *J* = 6.5 Hz, 2H, CH_2_), 3.62 (q, *J* = 7 Hz, 1H, CH), 4.23 (d, *J* = 5.5 Hz, 2H, CH_2_) 7.07–7.30 (m, 9H, Ar), 8.39 (t, *J* = 5.5 Hz, 1H, NH). IR (Nujol) 3311, 1645, 1546, 1466, 1378, 1230 cm^−1^. Elemental analysis: calculated for C_20_H_25_NO (295.43)% C 81.31; H 8.53; N 4.74; found % C 81.36; H 8.51; N 4.73.

### N-(4-Fluorobenzyl)-2–(4-isobutylphenyl)propanamide (4)

Obtained following the general procedure by the condensation between **1** and 4-fluorobenzylamine. Yield 83%. m.p. 58–61 °C. ^1^H NMR (DMSO-d_6_) δ 0.85 (d, *J* = 6.5 Hz, 6H, CH_3_), 1.34 (d, *J* = 7.0 Hz, 3H, CH_3_), 1.81 (hept, *J* = 6.5–7.0 Hz, 1H, CH), 2.41 (d, *J* = 6.5 Hz, 2H, CH_2_), 3.62 (q, *J* = 7.0 Hz, 1H, CH), 4.23 (d, *J* = 5.5 Hz, 2H, CH_2_) 7.04–7.22 (m, 8H, Ar), 8.39 (t, *J* = 5.5 Hz, 1H, NH). IR (Nujol) 3308, 1638, 1538, 1512, 1463 cm^−1^. Elemental analysis: calculated for C_20_H_24_FNO (313.42)% C 76.60; H 7.72; N 4.47; found % C 76.70; H 7.70; N 4.45.

### N-(4-Chlorobenzyl)-2–(4-isobutylphenyl)propanamide (5)

Obtained following the general procedure by the condensation between **1** and 4-chlorobenzylamine. Yield 82%. m.p. 65–68 °C. ^1^H NMR (DMSO-d_6_) δ 0.85 (d, *J* = 6.5 Hz, 6H, CH_3_), 1.34 (d, *J* = 7.3 Hz, 3H, CH_3_), 1.81 (hept, *J* = 6.5–7.0 Hz, 1H, CH), 2.41 (d, *J* = 6.5 Hz, 2H, CH_2_), 3.62 (q, *J* = 7.0 Hz, 1H, CH), 4.23 (d, *J* = 5.5 Hz, 2H, CH_2_) 7.07–7.30 (m, 8H, Ar), 8.42 (t, *J* = 5.0 Hz, 1H, NH). IR (Nujol) 3270, 3084, 1904, 1709, 1646, 1560, 1463, 1422 cm^−1^. Elemental analysis: calculated for C_20_H_24_ClNO (329.87)% C 72.82; H 7.33; N 4.25; found % C 72.88; H 7.32; N 4.24.

### N-(4-(tert-Butyl)benzyl)-2–(4-isobutylphenyl)propanamide (6)

Obtained following the general procedure by the condensation between **1** and 4-(tertbuthyl)benzylamine. Yield 80%. Oil. ^1^H NMR (DMSO-d_6_) δ 0.85 (d, *J* = 6.5 Hz, 6H, CH_3_), 1.23 (s, 9H, CH_3_) 1.34 (d, *J* = 7.0 Hz, 3H, CH_3_), 1.81 (m, 1H, CH), 2.41 (d, *J* = 6.5 Hz, 2H, CH_2_), 3.62 (q, *J* = 7.0 Hz, 1H, CH), 4.23 (d, *J* = 5.5 Hz, 2H, CH_2_) 7.07–7.26 (m, 8H, Ar), 8.39 (t, *J* = 5.5 Hz, 1H, NH). IR (Film) 3291, 1649, 1547, 1514 cm^−1^. Elemental analysis: calculated for C_24_H_33_NO (351.53)% C 82.00; H 9.46; N 3.98; found % C 82.07; H 9.44; N 3.97.

### 2–(4-Isobutylphenyl)-N-(4-(trifluoromethyl)benzyl)propanamide (7)

Obtained following the general procedure by the condensation between **1** and 4-(trifluoromethyl)benzylamine. Yield 82%. m.p. 62–64 °C. ^1^H NMR (DMSO-d_6_) δ 0.85 (d, *J* = 6.5 Hz, 6H, CH_3_), 1.34 (d, *J* = 7.0 Hz, 3H, CH_3_), 1.81 (hept, *J* = 6.5–7.0 Hz, 1H, CH), 2.41 (d, *J* = 6.5 Hz, 2H, CH_2_), 3.62 (q, *J* = 7.0 Hz, 1H, CH), 4.23 (d, *J* = 5.5 Hz, 2H, CH_2_) 7.07–7.60 (m, 8H, Ar), 8.49 (t, *J* = 5.5 Hz, 1H, NH). IR (Nujol) 3332, 3274, 1639, 1541, 1462 cm^−1^. Elemental analysis: calculated for C_21_H_24_F_3_NO (363.42)% C 69.40; H 6.66; N 3.85; found % C 69.48; H 6.64; N 3.83.

### 2–(4-Isobutylphenyl)-N-(4-methoxybenzyl)propanamide (8)

Obtained following the general procedure by the condensation between **1** and 4-methoxybenzylamine. Yield 81%. m.p. 78–80 °C. ^1^H NMR (DMSO-d_6_) δ 0.85 (d, *J* = 6.5 Hz, 6H, CH_3_), 1.34 (d, *J* = 7.0 Hz, 3H, CH_3_), 1.81 (hept, *J* = 6.5–7.0 Hz, 1H, CH), 2.41 (d, *J* = 6.5 Hz, 2H, CH_2_), 3.62 (q, *J* = 7.0 Hz, 1H, CH), 3.68 (s, 3H, OCH_3_) 4.23 (d, *J* = 5.5 Hz, 2H, CH_2_) 6.08–7.23 (m, 8H, Ar), 8.31 (t, *J* = 5.5 Hz, 1H, NH). IR (Nujol) 3284, 2360, 1710, 1648, 1462 cm^−1^. Elemental analysis: calculated for C_21_H_27_NO_2_ (325,45)% C 77.50; H 8.36; N 4.30; found % C 77.56; H 8.34; N 4.28.

### 2–(4-Isobutylphenyl)-N-(3-(trifluoromethyl)benzyl)propanamide (9)

Obtained following the general procedure by the condensation between **1** and 3-(trifluoromethyl)benzylamine. Yield 83%. m.p. 53–55 °C. ^1^H NMR (DMSO-d_6_) δ 0.85 (d, *J* = 6.5 Hz, 6H, CH_3_), 1.34 (d, *J* = 7.0 Hz, 3H, CH_3_), 1.81 (hept, *J* = 6.5–7.0 Hz, 1H, CH), 2.41 (d, *J* = 6.5 Hz, 2H, CH_2_), 3.62 (q, *J* = 7.0 Hz, 1H, CH), 4.23 (d, *J* = 5.5 Hz, 2H, CH_2_) 7.04–7.41 (m, 8H, Ar), 8.35 (t, *J* = 5.5 Hz, 1H, NH). IR (Nujol) 3288, 3073, 1651, 1584, 1452, 1329, 1165 cm^−1^. Elemental analysis: calculated for C_21_H_24_F_3_NO (363.42)% C 69.40; H 6.66; N 3.85; found % C 69.47; H 6.64; N 3.84.

### 2–(4-Isobutylphenyl)-N-(2-methoxybenzyl)propanamide (10)

Obtained following the general procedure by the condensation between **1** and 2-methoxybenzylamine. Yield 82%. m.p. 61–63 °C. ^1^H NMR (DMSO-d_6_) δ 0.85 (d, *J* = 6.5 Hz, 6H, CH_3_), 1.34 (d, *J* = 7.0 Hz, 3H, CH_3_), 1.81 (hept, *J* = 6.5–7.0 Hz, 1H, CH), 2.41 (d, *J* = 6.5 Hz, 2H, CH_2_), 3.62 (q, *J* = 7.0 Hz, 1H, CH), 3.68 (s, 3H, OCH_3_) 4.23 (d, *J* = 5.5 Hz, 2H, CH_2_) 6.75–7.24 (m, 8H, Ar), 8.15 (t, *J* = 5.5 Hz, 1H, NH). IR (Nujol) 3275, 1777, 1641, 1564, 1462, cm^−1^. Elemental analysis: Calculated for C_21_H_27_NO_2_ (325.45)% C 77.50; H 8.36; N 4.30; found % C 77.57; H 8.34; N 4.28.

### N-(2-Chlorobenzyl)-2–(4-isobutylphenyl)propanamide (11)

Obtained following the general procedure by the condensation between **1** and 2-chlorobenzylamine. Yield 85%. m.p. 60–63 °C. ^1^H NMR (DMSO-d_6_) δ 0.85 (d, *J* = 6.5 Hz, 6H, CH_3_), 1.34 (d, *J* = 7.0 Hz, 3H, CH_3_), 1.81 (hept, *J* = 6.5–7.0 Hz, 1H, CH), 2.41 (d, *J* = 6.5 Hz, 2H, CH_2_), 3.62 (q, *J* = 7.0 Hz, 1H, CH), 4.23 (d, *J* = 5.5 Hz, 2H, CH_2_) 7.08–7.04 (m, 8H, Ar), 8.39 (t, *J* = 5.5 Hz, 1H, NH). IR (Nujol): 3270, 1710, 1666, 1641, 1562 cm^−1^. Elemental analysis: calculated for C_20_H_24_ClNO (329.87)% C 72.82; H 7.33; N 4.25; found % C 72.88; H 7.32; N 4.24.

### N-(3-Hydroxy-4-methoxybenzyl)-2–(4-isobutylphenyl)propanamide (12)

Obtained following the general procedure by the condensation between **1** and 3-hydroxy-4-methoxybenzylamine. Yield 80%. m.p. 82–85 °C. ^1^H NMR (DMSO-d_6_) δ 0.85 (d, *J* = 6.5 Hz, 6H, CH_3_), 1.34 (d, *J* = 7.0 Hz, 3H, CH_3_), 1.81 (hept, *J* = 6.5–7.0 Hz, 1H, CH), 2.41 (d, *J* = 6.5 Hz, 2H, CH_2_), 3.62 (q, *J* = 7.0 Hz, 1H, CH), 3.72 (s, 3H, OCH_3_) 4.23 (d, *J* = 5.5 Hz, 2H, CH_2_) 6.62–7.21 (m, 7H, Ar), 8.39 (t, *J* = 5.5 Hz, 1H, NH) 9.45 (s, 1H, OH). IR (Nujol) 3334, 3276, 1642, 1564, 1462 cm^−1^. Elemental analysis: calculated for C_21_H_27_NO_3_ (341.45)% C 73.87; H 7.97; N 4.10; found % C 73.90; H 7.95; N 4.08.

### N-(3,4-Dichlorobenzyl)-2–(4-isobutylphenyl)propanamide (13)

Obtained following the general procedure to by the condensation between **1** and 3,4-dichlorobenzylamine. Yield 83%. m.p. 78–82 °C. ^1^H NMR (DMSO-d_6_) δ 0.85 (d, *J* = 6.5 Hz, 6H, CH_3_), 1.34 (d, *J* = 7.0 Hz, 3H, CH_3_), 1.81 (hept, *J* = 6.5–7.0 Hz, 1H, CH), 2.41 (d, *J* = 6.5 Hz, 2H, CH_2_), 3.62 (q, *J* = 7.0 Hz, 1H, CH), 4.23 (d, *J* = 5.5 Hz, 2H, CH_2_), 7.07–7.60 (m, 7H, Ar), 8.39 (t, *J* = 5.5 Hz, 1H, NH). IR (Nujol) 3268, 3072, 1647, 1549, 1428 cm^−1^. Elemental analysis: calculated for C_20_H_23_Cl_2_NO (364.31)% C 65.94; H 6.36; N 3.84; found % C 66.03; H 6.35; N 3.82.

### N-(2,4-Dichlorobenzyl)-2–(4-isobutylphenyl)propanamide (14)

Obtained following the general procedure by the condensation between **1** and 2,4-dichlorobenzylamine. Yield 79%. m.p. 73–75 °C. ^1^H NMR (DMSO-d_6_) δ 0.85 (d, *J* = 6.5 Hz, 6H, CH_3_), 1.34 (d, *J* = 7.0 Hz, 3H, CH_3_), 1.81 (hept, *J* = 6.5–7.0 Hz, 1H, CH), 2.41 (d, *J* = 6.5 Hz, 2H, CH_2_), 3.62 (q, *J* = 7.0 Hz, 1H, CH), 4.23 (d, *J* = 5.5 Hz, 2H, CH_2_) 7.07–7.60 (m, 7H, Ar), 8.39 (t, *J* = 5.5 Hz, 1H, NH). IR (Nujol) 3274, 3083, 1646, 1557 cm^−1^. Elemental analysis: calculated for C_20_H_23_Cl_2_NO (364.31)% C 65.94; H 6.36; N 3.84; found % C 66.03; H 6.35; N 3.82.

### N-(2,5-Dichlorobenzyl)-2–(4-isobutylphenyl)propanamide (15)

Obtained following the general procedure by the condensation between **1** and 2,5-dichlorobenzylamine. Yield 82%. m.p. 93–96 °C. ^1^H NMR (DMSO-d_6_) δ 0.85 (d, *J* = 6.5 Hz, 6H, CH_3_), 1.34 (d, *J* = 7.3 Hz, 3H, CH_3_), 1.81 (hept, *J* = 6.5–7.0 Hz, 1H, CH), 2.41 (d, *J* = 6.5 Hz, 2H, CH_2_), 3.62 (q, *J* = 7.0 Hz, 1H, CH), 4.23 (d, *J* = 5.5 Hz, 2H, CH_2_) 7.06–7.40 (m, 7H, Ar), 8.39 (t, *J* = 5.5 Hz, 1H, NH). IR (Nujol) 3268, 3072, 1647, 1549, 1428 cm^−1^. Elemental Analysis: calculated for C_20_H_23_Cl_2_NO (363.42)% C 65.94; H 6.36; N 3.84; found % C 66.03; H 6.35; N 3.82.

### N-(2,6-Dichlorobenzyl)-2–(4-isobutylphenyl)propanamide (16)

Obtained following the general procedure by the condensation between **1** and 2,6-dichlorobenzylamine. Yield 82%. m.p. 130–135 °C. ^1^H NMR (DMSO-d_6_) δ 0.85 (d, *J* = 6.5 Hz, 6H, CH_3_), 1.34 (d, *J* = 7.0 Hz, 3H, CH_3_), 1.81 (hept, *J* = 6.5–7.0 Hz, 1H, CH), 2.41 (d, *J* = 6.5 Hz, 2H, CH_2_), 3.62 (q, *J* = 7.0 Hz, 1H, CH), 4.23 (d, *J* = 5.5 Hz, 2H, CH_2_) 7.04–7.45 (m, 7H, Ar), 8.39 (t, *J* = 5.5 Hz, 1H, NH). IR (Nujol) 3310, 1641, 1534, 1437 cm^−1^. Elemental analysis: calculated for C_20_H_23_Cl_2_NO (363.42)% C 65.94; H 6.36; N 3.84; found % C 66.00; H 6.35; N 3.82.

### General procedure for the synthesis of phenylpiperazine derivatives 18–27

A solution of **1** (0.21 g, 1 mmol), EDC (0.19 g, 1.1 mmol) and HOBt (0.13 g, 1 mmol) in anhydrous MeCN (10 ml) was stirred at r.t. for 30 min, then the appropriate arylpiperazine **17** (1 mmol) was added. The mixture was then stirred for 12 h at r.t. After the solvent was removed under vacuum, the residue was dissolved in AcOEt (20 ml) and washed sequentially with brine (2 × 5 ml), 10% citric acid (2 × 5 ml), NaHCO_3_ 10% aqueous solution (2 × 5 ml) and water (2 × 5 ml). The organic layer was dried over anhydrous Na_2_SO_4_ and evaporated under vacuum. The obtained residue was tritured with iPr_2_O; the precipitate was then filtrated to obtain the compounds **18–27.**

### 2–(4-Isobutylphenyl)-1–(4-phenylpiperazin-1-yl)propan-1-one (18)

Obtained following the general procedure by the condensation between **1** and 1-phenylpiperazine. Yield 97%. m.p. 75–80 °C. ^1^H NMR (DMSO-d_6_) δ 0.83 (d, *J* = 7.0 Hz, 6H, CH_3_), 1.30 (d, *J* = 7.0 Hz, 3H, CH_3_), 1.80 (hept, *J* = 7.0 Hz, 1H, CH), 2.42 (d, *J* = 7.0 Hz, 2H, CH_2_), 3.16 (m, 2H, CH_2_), 3.20 (m, 2H, CH_2_), 3.40 (m, 1H, CH), 3.48–3.65 (m, 4H, CH_2_), 6.81 (m, 1H Ar), 7.04–7.45 (m, 6H, Ar), 7.53 (m, 1H Ar), 7.59 (m, 1H Ar). IR (Nujol) 3273, 1741, 1631, 1600, 1508, 1465 cm^−1^. Elemental analysis: calculated for C_23_H_30_N_2_O (350.51)% C 78.82; H 8.63; N 7.99; found % C 78.89; H 8.67; N 7.85.

### 1–(4-(3-Chlorophenyl)piperazin-1-yl)-2–(4-isobutylphenyl)propan-1-one (19)

Obtained following the general procedure by the condensation between **1** and 1–(3-chlorophenyl)piperazine. Yield 95%. Oil. ^1^H NMR (DMSO-d_6_) δ 0.84 (d, *J* = 6.5 Hz, 6H, CH_3_), 1.32 (d, *J* = 7.0 Hz, 3H, CH_3_), 1.81 (hept, *J* = 6.5–7.0 Hz, 1H, CH), 2.43 (d, *J* = 7.0 Hz, 2H, CH_2_), 2.89 (m, 2H, CH_2_), 3.23 (m, 2H, CH_2_), 3.41 (q, *J* = 7.0 Hz, 1H, CH), 3.45–3.68 (m, 4H, CH_2_), 6.70 (m, 1H Ar), 7.06–7.43 (m, 6H, Ar), 7.50 (m, 1H Ar). IR (Film) 3437, 1732, 1646, 1594, 1486, 1463, 1384, 1231 cm^−1^. Elemental analysis: calculated for C_23_H_29_ClN_2_O (384.95)% C 71.76; H 7.59; N 7.28; found % C 71.75; H 7.60; N 7.35.

### 1–(4-(4-Chlorophenyl)piperazin-1-yl)-2–(4-isobutylphenyl)propan-1-one (20)

Obtained following the general procedure by the condensation between **1** and 1–(4-chlorophenyl)piperazine. Yield 83%. Oil. ^1^H NMR (DMSO-d_6_) δ 0.94 (d, *J* = 7.0 Hz, 6H, CH_3_), 1.32 (d, *J* = 6.5 Hz, 3H, CH_3_), 1.81 (hept, *J* = 7.0 Hz, 1H, CH), 2.66 (m, 2H, CH_2_), 2.95 (m, 2H, CH_2_), 3.11 (m, 2H, CH_2_), 3.50–3.68 (m, 4H, CH_2_), 4.17 (q, *J* = 6.5 Hz, 1H, CH), 6.89 (m, 1H, Ar), 7.03 (m, 1H, Ar), 7.16–7.21 (m, 5H, Ar), 7.30 (m, 1H, Ar). IR (Film) 3421, 2955, 1731, 1645, 1497, 1463, 1384 cm^−1^. Elemental analysis: calculated for C_23_H_29_ClN_2_O (384.95)% C 71.76; H 7.59; N 7.28; found % C 71.70; H 7.65; N 7.30.

### 1–(4-(3,4-Dichlorophenyl)piperazin-1-yl)-2–(4-isobutylphenyl)propan-1-one (21)

Obtained following the general procedure by the condensation between **1** and 1–(3,4-dichlorophenyl)piperazine. Yield 90%. Oil. ^1^H NMR (DMSO-d_6_) δ 0.86 (d, *J* = 6.5 Hz, 6H, CH_3_), 1.33 (d, *J* = 7.0 Hz, 3H, CH_3_), 1.80 (hept, *J* = 6.5–7.0 Hz, 1H, CH), 2.41 (d, *J* = 7.0 Hz, 2H, CH_2_), 2.92 (m, 2H, CH_2_), 3.17 (m, 2H, CH_2_), 3.22 (m, 1H, CH), 3.40–3.71 (m, 4H, CH_2_), 6.88 (m, 1H Ar), 7.06–7.43 (m, 5H, Ar), 7.53 (m, 1H Ar). IR (Film) 3433, 1728, 1645, 1594, 1555, 1484, 1230 cm^−1^. Elemental analysis: calculated for C_23_H_28_Cl_2_N_2_O (419.39)% C 64.87; H 6.73; N 6.68; found % C 64.78; H 6.68; N 6.59.

### 1–(4-(4-Fluorophenyl)piperazin-1-yl)-2–(4-isobutylphenyl)propan-1-one (22)

Obtained following the general procedure by the condensation between **1** and 1–(4-fluorophenyl)piperazine. Yield 97%. m.p. 45–50 °C. ^1^H NMR (DMSO-d_6_) δ 0.90 (d, *J* = 7.0 Hz, 6H, CH_3_), 1.36 (d, *J* = 6.5 Hz, 3H, CH_3_), 1.87 (hept, *J* = 6.5–7.0 Hz, 1H, CH), 2.61 (m, 2H, CH_2_), 2.99 (m, 2H, CH_2_), 3.06 (m, 2H, CH_2_), 3.44–3.61 (m, 4H, CH_2_), 4.19 (q, *J* = 6.5 Hz, 1H, CH), 6.97 (m, 1H, Ar), 7.02 (m, 1H, Ar), 7.10–7.27 (m, 5H, Ar), 7.34 (m, 1H, Ar). IR (Nujol) 3445, 2955, 2929, 1644, 1510, 1441, 1230 cm^−1^. Elemental analysis: calculated for C_23_H_29_FN_2_O (368.23)% C 74.97; H 7.93; N 7.60; found % C 75.01; H 7.90; N 7.55.

### 2–(4-Isobutylphenyl)-1–(4-(4-methoxyphenyl)piperazin-1-yl)propan-1-one (23)

Obtained following the general procedure by the condensation between **1** and 1–(4-methoxyphenyl)piperazine. Yield 95%. Oil. ^1^H NMR (DMSO-d_6_) δ 0.95 (d, *J* = 7.0 Hz, 6H, CH_3_), 1.30 (d, *J* = 6.5 Hz, 3H, CH_3_), 1.83 (hept, *J* = 6.5–7.0 Hz, 1H, CH), 2.62 (m, 2H, CH_2_), 3.03 (m, 2H, CH_2_), 3.09 (m, 2H, CH_2_), 3.40–3.59 (m, 4H, CH_2_), 3.66 (s, 3H, CH_3_), 4.22 (q, *J* = 6.5 Hz, 1H, CH), 7.01 (m, 1H, Ar), 7.04 (m, 1H, Ar), 7.12–7.23 (m, 5H, Ar), 7.38 (m, 1H, Ar). IR (Film) 3440, 2954, 2930, 1732, 1644, 1464, 1442, 1246 cm^−1^. Elemental analysis: calculated for C_24_H_32_N_2_O_2_ (380.53)% C 75.75; H 8.48; N 7.36; found % C 75.80; H 8.53; N 7.30.

### 2–(4-Isobutylphenyl)-1–(4-(3-methoxyphenyl)piperazin-1-yl)propan-1-one (24)

Obtained following the general procedure by the condensation between **1** and 1–(3-methoxyphenyl)piperazine. Yield 92%. Oil. ^1^H NMR (DMSO-d_6_) δ 0.82 (d, *J* = 7.0 Hz, 6H, CH_3_), 1.28 (d, *J* = 6.0 Hz, 3H, CH_3_), 1.79 (hept, *J* = 6.0–7.0 Hz, 1H, CH), 2.87 (m, 2H, CH_2_), 3.01 (m, 2H, CH_2_), 3.13 (m, 2H, CH_2_), 3.44–3.54 (m, 4H, CH_2_), 3.68 (s, 3H, CH_3_), 4.08 (q, *J* = 6.5 Hz, 1H, CH), 6.34 (m, 2H, Ar), 6.42 (m, 1H, Ar), 7.05–7.10 (m, 3H, Ar), 7.16 (m, 2H, Ar). IR (Film) 2956, 2928, 1734, 1647, 16071460,1203 cm^−1^. Elemental analysis: calculated for C_24_H_32_N_2_O_2_ (380.53)% C 75.75; H 8.48; N 7.36; found % C 75.70; H 8.50; N 7.34.

### 2–(4-Isobutylphenyl)-1–(4-(m-tolyl)piperazin-1-yl)propan-1-one (25)

Obtained following the general procedure by the condensation between **1** and 1–(3-methylphenyl)piperazine. Yield 91%. Oil. ^1^H NMR (DMSO-d_6_) δ 0.81 (d, *J* = 7.0 Hz, 6H, CH_3_), 1.27 (d, *J* = 6.0 Hz, 3H, CH_3_), 1.78 (hept, *J* = 6.0–7.0 Hz, 1H, CH), 2.20 (s, 3H, CH_3_), 2.50 (d, *J* = 7.0 Hz, 2H, CH_2_), 2.83 (m, 1H, CH_2_), 2.98 (m, 1H, CH_2_), 3.13 (m, 1H, CH_2_), 3.46–3.53 (m, 4H, CH_2_), 3.73 (m, 1H, CH_2_), 4.09 (q, *J* = 6.5 Hz, 1H, CH), 6.58–6.64 (m, 3H, Ar), 7.05 (m, 1H, Ar), 7.09 (d, *J* = 8.0, 2H, Ar), 7.16 (d, *J* = 8.0 Hz, 2H, Ar). IR (Film) 3483, 2955, 1926, 1644, 1602, 1494, 1434, 1233,1185 cm^−1^. Elemental analysis: calculated for C_24_H_32_N_2_O (364.25)% C 79.08; H 8.85; N 7.68; found % C 79.15; H 8.92; N 7.60.

### 1–(4-(2,3-Dimethylphenyl)piperazin-1-yl)-2–(4-isobutylphenyl)propan-1-one (26)

Obtained following the general procedure by the condensation between **1** and 1–(2,3-dimethylphenyl)piperazine. Yield 90%. Oil. ^1^H NMR (DMSO-d_6_) δ 0.83 (d, *J* = 7.0 Hz, 6H, CH_3_), 1.30 (d, *J* = 7.0 Hz, 3H, CH_3_), 1.78 (hept, *J* = 7.0 Hz, 1H, CH), 2.12 (m, 3H, CH_3_), 2.18 (m, 3H, CH_3_), 2.40 (m, 2H, CH_2_) 2.12 (m, 2H, CH_2_), 2.50 (m, 2H, CH_2_), 2.72 (m, 2H, CH_2_), 3.56 (m, 2H, CH_2_), 4.09 (q, *J* = 7 Hz, 1H, CH), 6.76 (m, 1H, Ar), 6.69 (m, 1H, Ar), 6.99 (m, 1H, Ar), 7.11 (m, 2H, Ar), 7.17 (m, 2H, Ar). IR (Film) 2959, 1731, 1644, 15111471, 1367, 1235 cm^−1^. Elemental analysis: calculated for C_25_H_34_N_2_O (378.56)% C 79.32; H 9.05; N 7.40; found % C 79.28; H 9.02; N 7.50

### 2–(4-Isobutylphenyl)-1–(4-(o-tolyl)piperazin-1-yl)propan-1-one (27)

Obtained following the general procedure by the condensation between **1** and 1–(2-methylphenyl)piperazine. Yield 91%. Oil. ^1^H NMR (DMSO-d_6_) δ 0.83 (d, *J* = 6.5 Hz, 6H, CH_3_), 1.29 (d, *J* = 7.0 Hz, 3H, CH_3_), 1.81 (hept, *J* = 6.5–7.0 Hz, 1H, CH), 2.21 (s, 3H, CH_3_), 2.24 (m, 1H, CH_2_), 2.41 (d, *J* = 6.0 Hz, 2H, CH_2_), 2.63 (m, 2H, CH_2_), 2.76 (m, 1H, CH_2_), 3.43 (m, 1H, CH_2_), 3.56 (m, 2H, CH_2_), 3.69 (m, 1H, CH_2_), 4.10 (q, *J* = 7.0 Hz, 1H, CH), 6.82 (m, 1H, Ar), 6.93 (m, 1H, Ar), 7.07–7.14 (m, 4H, Ar), 7.42 (d, *J* = 7.5, 2H, Ar). IR (Film) 2923, 1639, 1600, 1494, 14631377,1224, 1147 cm^−1^. Elemental analysis: calculated for C_25_H_32_N_2_O (364.25)% C 79.32; H 8.85; N 7.68; found % C 79.27; H 9.00; N 7.59.

### 2–(4-Isobutylphenyl)-1-(piperazin-1-yl)propan-1-one trifluoroacetate (29)

Compound **1** (2.06 g, 10 mmol), EDC (2.09 g, 11 mmol) and HOBt (1.35 g, 10 mmol) were dissolved in MeCN (10 ml). The mixture was stirred at r.t. for 30 min, then BOC-piperazine (**28**) (1.86 g, 10 mmol) was added. The mixture was stirred at r.t. for 12 h. After the solvent was removed under vacuum. The residue was dissolved in AcOEt (20 ml) and washed sequentially with brine (2 × 5 ml), 10% citric acid (2 × 5 ml), saturated NaHCO_3_ aqueous solution (2 × 5 ml) and water (2 × 5 ml). The organic layer was dried over anhydrous Na_2_SO_4_ and evaporated under vacuum. The obtained residue was dissolved in dichloromethane, without further purification, added trifluoroacetic acid (TFA) (20 ml) and stirred at r.t. for 24 h to obtain the BOC de-protected compound. Then the solvent was removed under vacuum and to the obtained residue diethyl ether (Et_2_O) (20 ml) was added leading to formation of a solid that was filtered to give the title compound. Yield 97%. Oil. ^1^H NMR (DMSO-d_6_) δ 0.84 (d, *J* = 5.0 Hz, 6H, CH_3_), 1.28 (d, *J* = 5.0 Hz, 3H, CH_3_), 1.81 (m, 1H, CH), 2.41 (d, *J* = 6.0 Hz, 2H, CH_2_), 2.56 (m, 1H, CH_2_), 3.00 (m, 3H, CH_2_), 3.34 (m, 1H, CH_2_), 3.58 (m, 1H, CH_2_), 3.74 (m, 2H, CH_2_), 4.09 (m, 1H, CH), 7.11 (m, 2H, Ar), 7.18 (d, 2H, Ar), 8.88 (s, 1H, NH). IR (Film) 2957, 2925, 2854, 1674, 1636, 1461, 1442, 1367, 1199, 1082 cm^−1^. Elemental analysis: calculated for C_19_H_27_F_3_N_2_O_3_ (388.42)% C 58.75; H 7.01; N 7.21; found % C 58.80; H 6.99; N 7.17.

### General procedure for the synthesis of benzylpiperazine 30–34

To a solution of compound **29** (0.39 g, 1 mmol) in dichloromethane (CH_2_Cl_2_) (10 ml) the appropriate arylaldehyde (1.6 mmol), sodium sodium hydrogen carbonate (NaHCO_3_) (0.10 g, 1.2 mmol) and sodium triacetoxyborohydride (NaBHAc_3_) (0.32 g, 1.5 mmol) were added; the mixture was then stirred at r.t. for 24 h. After the mixture was basified to pH 10 with a solution of NaOH 0.1 N, then extracted with CH_2_Cl_2_ (3 × 20 ml). The organic phases were collected, dried over sodium Na_2_SO_4_, filtrated and the solvent removed to obtain the desired compound.

### 1–(4-Benzyl-1-yl)-2–(4-isobutylphenyl)propan-1-one (30)

Obtained following the general procedure by the reductive alkylation between **29** and benzaldehyde. Yield 91%. Oil. ^1^H NMR (DMSO-d_6_) δ 0.85 (d, *J* = 7.0 Hz, 6H, CH_3_), 1.25 (d, *J* = 7.0 Hz, 3H, CH_3_), 1.78 (m, 1H, CH_2_), 1.81 (q, *J* = 6.0 Hz, 1H, CH), 2.21 (m, 1H, CH_2_), 2.34 (m, 1H, CH_2_), 2.41 (d, *J* = 7.5 Hz, 2H, CH_2_), 2.50 (s, 1H, CH_2_), 3.36 (m, 3H, CH_2_), 3.38 (s, 2H, CH_2_), 3.60 (m, 1H, CH_2_), 4.03 (m, 1H, CH), 7.08 (m, 2H, Ar), 7.13 (d, 2H, Ar), 7.23 (m, 3H, Ar), 7.29 (d, 2H, Ar). IR (Film) 3448, 2954, 2929, 2645, 1462, 1230, 1032, 1000 cm^−1^. Elemental analysis: calculated for C_24_H_32_N_2_O (364.25)% C 79.08; H 8.85; N 7.68; found % C 79.15; H 8.80; N 7.65.

### 1–(4-(2-Chlorobenzyl)piperazin-1-yl)-2–(4-isobutylphenyl)propan-1-one (31)

Obtained following the general procedure by the reductive alkylation between **29** and 2-chlorobenzaldehyde. Yield 87%. Oil. ^1^H NMR (DMSO-d_6_) δ 0.83 (d, *J* = 7.0 Hz, 6H, CH_3_), 1.23 (d, *J* = 7.0 Hz, 3H, CH_3_), 1.79 (q, *J* = 6.0 Hz, 1H, CH), 2.40 (d, *J* = 7.5 Hz, 2H, CH_2_), 3.32–3.75 (m, 8H, CH_2_), 4.00 (m, 1H, CH), 4.56 (s, 2H, CH_2_), 6.99–7.56 (m, 8H, Ar). IR (Film) 3416, 2955, 2927, 1628, 1060, 1033 cm^−1^. Elemental analysis: calculated for C_24_H_31_ClN_2_O (398.98)% C 72.25; H 7.83; N 7.02; found % C 72.30; H 7.81; N 7.08.

### 1–(4-(3-Chlorobenzyl)piperazin-1-yl)-2–(4-isobutylphenyl)propan-1-one (32)

Obtained following the general procedure by the reductive alkylation between **29** and 3-chlorobenzaldehyde. Yield 90%. Oil. ^1^H NMR (DMSO-d_6_) δ 0.96 (d, *J* = 6.5 Hz, 6H, CH_3_), 1.36 (d, *J* = 7.5 Hz, 3H, CH_3_), 1.94 (q, *J* = 6.5–7.5 Hz, 1H, CH), 2.52 (d, *J* = 7.5 Hz, 2H, CH_2_), 3.37–3.85 (m, 8H, CH_2_), 4.14 (m, 1H, CH), 4.62 (s, 2H, CH_2_), 7.13–7.55 (m, 8H, Ar). IR (Film) 3414, 2955, 2928,1702, 1631, 1464, 1434, 1228, 1196 cm^−1^. Elemental analysis: calculated for C_24_H_31_ClN_2_O (398.98)% C 72.25; H 7.83; N 7.02; found % C 72.38; H 7.85; N 7.00.

### 2–(4-Isobutylphenyl)-1–(4-(4-(trifluoromethyl)benzyl)piperazin-1-yl)propan-1-one (33)

Obtained following the general procedure by the reductive alkylation between **29** and 4-(trifluoromethyl)benzaldehyde. Yield 50%. Oil. ^1^H NMR (DMSO-d_6_) δ 0.96 (d, *J* = 6.5 Hz, 6H, CH_3_), 1.37 (d, *J* = 6.5 Hz, 3H, CH_3_), 1.90 (q, *J* = 6.5 Hz, 1H, CH), 2.53 (s, 2H, CH_2_), 2.61 (d, *J* = 6.5 Hz, 2H, CH_2_), 3.43–3.70 (m, 8H, CH_2_), 4.12 (m, 1H, CH), 7.13–7.27 (m, 8H, Ar). IR (Film) 3332, 2955, 32868, 1644, 1510, 1462, 1367, 1228, 1164, 1125, 1066 cm^−1^. Elemental analysis: calculated for C_25_H_31_F_3_N_2_O (432.53)% C 69.42; H 7.22; N 6.48; found % C 69.48; H 7.21; N 6.53.

### 1–(4-(3-Fluorobenzyl)piperazin-1-yl)-2–(4-isobutylphenyl)propan-1-one (34)

Obtained following the general procedure by the reductive alkylation between **29** and 3-fluorobenzaldehyde. Yield 49%. Oil. ^1^H NMR (DMSO-d_6_) δ 0.96 (d, *J* = 6.5 Hz, 6H, CH_3_), 1.36 (d, *J* = 6.5 Hz, 3H, CH_3_), 1.89 (q, *J* = 6.5 Hz, 1H, CH), 2.51 (s, 2H, CH_2_), 2.61 (d, *J* = 6.5 Hz, 2H, CH_2_), 3.32–3.52 (m, 8H, CH_2_), 4.12 (m, 1H, CH), 7.15–7.25 (m, 8H, Ar). IR (Film) 3407, 2955, 2926, 1713, 1696, 1631, 1591, 1254, 1001 cm^−1^. Elemental analysis: calculated for C_24_H_31_FN_2_O (382.52)% C 75.36; H 8.17; N 7.32; found % C 75.40; H 8.05; N 7.40.

### Computational studies

#### FAAH receptor and ibuprofen amides preparation

The crystal structure of the fatty acid amide hydrolase (FAAH) (PDB ID: 3QK5)^27^ has been downloaded from the Protein Data Bank website. Both monomers A and B were treated with the Protein Preparation Wizard^28^ tool implemented in Maestro ver. 11.1^29^ in order to add all the hydrogen atoms and assign the correct bond orders. Moreover, the co-crystallised ligand (QK5), as well as all the crystallographic water molecules, were removed. Residue Lys142 was considered in its deprotonated form, according to the proposed catalytic mechanism^2^^,^^3^^,^^30^. The 3 D structure of amides described above was built using the Graphical User Interface of Maestro ver. 11.1^29^, as (*S*)-enantiomers in view of our data with (*S*)-**Ibu-AM5**, which was ten-fold more potent inhibitor than the (*R*)-enantiomer^23^. The protonation state of these amides at pH 7.4 in water has been calculated using the Epik module^31^, revealing the protonation of the nitrogen atom of the piperazine ring only of compounds **30–34** characterised by a methylene bridge between the aryl and the piperazine rings. Finally, each compound was then minimised with the OPLS_2005 force field using the Polak-Ribiere Conjugate Gradient (PRCG) algorithm and 2500 iteration steps.

#### Induced-Fit docking protocol

Only the monomer A of the rat FAAH (*r*FAAH) receptor was considered for the induced-fit docking (IFD)^32^^,^^33^. The IFD protocol has three stages. In the first stage, ligands were docked to rigid protein using initial Glide softened potential (van der Waals radii scaling). The top 20 poses for each ligand were retained. In the second stage, a Prime side-chain prediction for each protein/ligand complex on residues within a default distance of 5 Å was performed followed by a Prime minimisation of the same set of residues and protein/ligand complexes. In the third stage, a Glide re-docking of each protein/ligand complex within a specified default lowest-energy structure (30 kcal/mol) was carried out. In this step, each ligand is rigorously docked using the default Glide settings, into the induced-fit receptor structure. At the end of the final stage, two methods were used for estimation of the binding energy for each output complex pose (IFDScore and Glide_emodel). Glide SP (Standard Precision) was used for all docking calculations. Docking poses were ranked on the basis of Glide_emodel energy. A preliminary validation of the computational protocol was performed by reproducing with IFD the crystallographic binding mode of QK5 ligand complexed in the model PDB ID: 3QK5 (QK5) (Supplementary Figure S2-A)^27^ and that of the (*S*)-**Ibu-AM5** found after Molecular Dynamics simulations^23^ (Supplementary Figure S2-B).

### Pharmacology

#### FAAH assay

Frozen (−80 °C) brains (minus cerebella) from adult Wistar or Sprague-Dawley rats were thawed and homogenised in 20 mM HEPES, 1 mM MgCl_2_, pH 7.0. and thereafter centrifuged at ∼35000 × g for 20 min at 4 °C. Homogenates were washed (by centrifugation at ∼35000 × g for 20 min at 4 °C followed by resuspension in the buffer) twice and incubated at 37 °C for 15 min in order to hydrolyse all endogenous FAAH substrates. After a further centrifugation, pellets were resuspended in 50 mM Tris-HCl buffer, pH 7.4, containing 1 mM EDTA and 3 mM MgCl_2_, and frozen at −80 °C in aliquots until used for the assay. For the FAAH assay^34^ test compounds, homogenates (usually 0.5–0.8 μg protein per assay, diluted with 10 mM Tris-HCl, 1 mM EDTA pH 7.4) and 25 μL of [^3^H]AEA in 10 mM Tris- HCl, 1 mM EDTA, pH 7.4, containing 1% w/v fatty acid-free bovine serum albumin, final substrate concentration of 0.5 μM) were incubated for 10 min at 37 °C (final assay volume 200 μL). Reactions were stopped by placing the tubes on ice. Final assay concentrations of the solvents used for the compounds (ethanol or DMSO) were in the range 1–5%. Activated charcoal (80 μL + 320 μL 0.5 M HCl) was added and the samples were mixed and left at room temperature for about 30 min. Following centrifugation at 2500 rpm for 10 min, aliquots (200 μL) of the supernatants were analysed for tritium content by liquid scintillation spectroscopy with quench correction. Blank values were obtained by the use of buffer rather than homogenate.

In general, FAAH assays upon three homogenates were undertaken using separate inhibitor dilution series (from a stock solution), with 6 concentrations of inhibitor in half-log concentrations (i.e. 1, 3, 10 µM etc) ranging from 0.3–100 µM. Data were expressed as % of vehicle control and analysed using the algorithm log(inhibitor) vs. response – variable slope (four parameters) built into the GraphPad Prism computer programme for the Macintosh (GraphPad Software Inc., San Diego, CA). Two different curve fits were chosen: one where the top (uninhibited) value was set to 100 and the bottom (maximum inhibition) was set to 0, and one where the top was set to 100 and the bottom allowed to float. The best model (when the bottom value returned >0) was chosen by Akaike’s informative criteria. Since the programme uses log_10_ inhibitor concentrations, the IC_50_ values for the inhibitable fraction are derived from the corresponding − log_10_(IC_50_) (pI_50_) values. Hence the SE values are for the pI_50_ values rather than the IC_50_ values. In consequence, we report both pI_50_ and IC_50_ values to indicate the SE values.

## Results and discussion

**Scheme 1.**
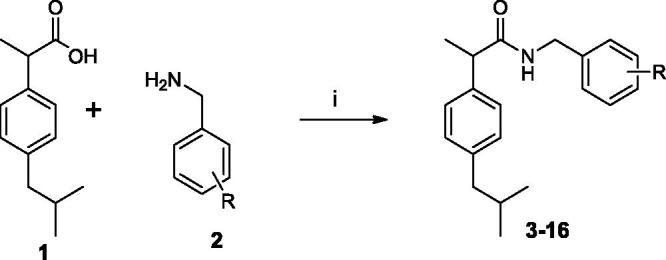
Synthesis of Ibuprofen amides **3–16**. (i) EDC, OH-Bt, MeCN, r.t. 24 h.

**Scheme 2.**
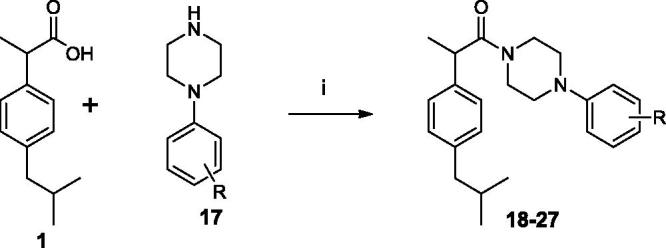
Synthesis of Ibuprofen amides **18–27**. (i) EDC, OH-Bt, MeCN, r.t. 12 h.

**Scheme 3.**
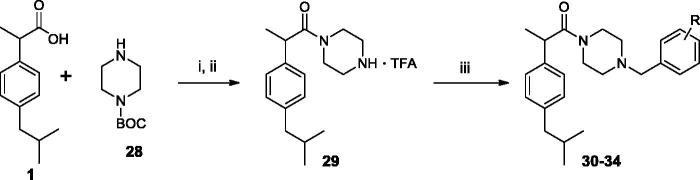
Synthesis of Ibuprofen amides **30–34**. (i) EDC, OH-Bt, MeCN, r.t. 12 h, (ii) TFA, CH2Cl2, r.t., 24 h.; (iii) NaBHAc3, NaHCO3, ArCHO, r.t., 24 h.

The target amides of ibuprofen were synthesised according to the Schemes 1–3. The benzylamides were obtained by coupling ibuprofen (**1**) with substituted benzylamines (**2**) in the presence of EDC and HOBt in MeCN solution. This synthetic pathway was found to be clean and high yielding.

The benzylamides **3–16**, along with the reference compound **Ibu-AM5**, were evaluated for their ability to inhibit FAAH. The inhibition assays were performed using 0.5 μM [^3^H]AEA as substrate and rat brain homogenates as the enzyme source. For comparison, the % inhibition produced by URB597 (which shows time-dependent inhibition) using this assay in our laboratory but following a 60 min preincubation, was 8, 38, 98 and 100 at concentrations of 0.1, 1, 10 and 100 nM, respectively^35^. The results of these primary assays are shown in Table 1. The replacement of the **Ibu-AM5** 2-methylpyridine moiety with a benzyl moiety to give benzylamides **3–16** led to a reduction in inhibitory activity. In general, benzylamide derivatives showed IC_50_ values ranging 18–51 µM with the exception of **11**, and **15**. Compounds **11** and **15** showed FAAH inhibitory activity with IC_50_ values of 4.1 and 4.4 µM, respectively (Table 1). As shown in Table 1, the position and the kind of substituent on benzyl moiety affect the inhibitory activity. The presence of 4-substituent is not favourable for the inhibitory activity. Conversely, the presence of substituents at 2-position improved the activity as compared with the unsubstituted amide **3**, as showed by the 2-chloro (**11**) and 2,5-dichloro (**15**) derivatives. Moreover, the moving of chlorine atom from 5-position of amide **15** to 4-position to give the analogue **14** led to a reduction in activity (IC_50_ 21 µM). While the moving of a chlorine atom from 5-position to 6-position (amide **16**) led to a complete loss of activity.

**Figure 2.**
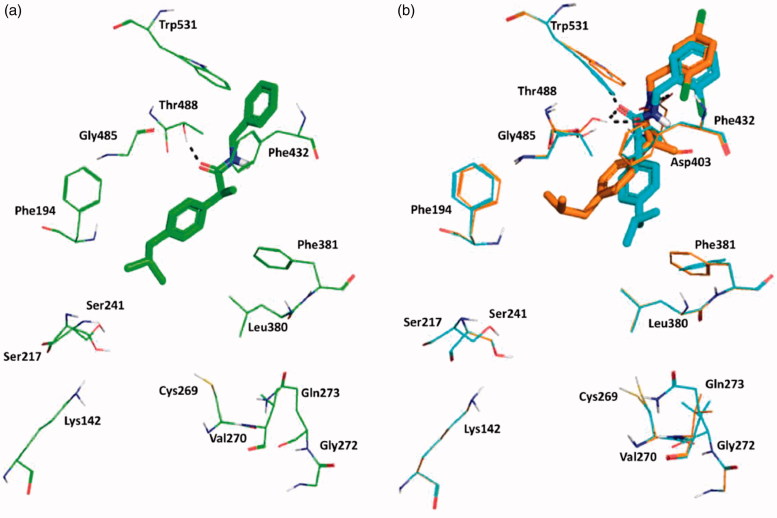
The lowest emodel binding modes of (S)-benzylamides : (a) **3**, (b) **11** and **15**. Key interacting residues of FAAH are displayed as green, cyan and orange lines relatively to benzylamides **3**, **11**, and **15** that are represented as green, cyan and orange sticks, respectively. Hydrogen bond interactions detected by Maestro 11.1 are shown as dashed black lines.

We also studied the binding of the newly designed compounds in the active site of FAAH by using the Induced fit protocol (IFD) implemented in Maestro (Schrödinger), that takes into consideration not only ligand flexibility, but also protein rearrangements upon ligand binding. Calculations were carried out in the monomer A of the rat FAAH (*r*FAAH) (PDB ID: 3QK5). Taking into considerations previous results on stereoselectivity of Ibu-AM5, demonstrating (*S*)-IbuAM5 being 10-fold more active than (*R*)-Ibu-AM5^23^, we performed and discussed docking calculations on the (*S*)-enantiomers of selected compounds in the series. However, for the sale of completeness, docking results for the (*R*)-enantiomers were reported as Supplementary information. A preliminary validation of the computational protocol was performed on (*S*)-**Ibu-AM5** and QK5 ligand. Results highlighted that the pose with the lowest Glide-emodel and IFDScore reproduced closely the binding mode of (*S*)-**Ibu-AM5** and QK5 (RMSD: 1.34 Å and 0.58 Å respectively; (Supplementary Figure S1). We have compared the binding mode of amides **3**, **11** and **15**. Best poses of compounds **3** (Figure 2(a)), **11** and **15** (Figure 2(b)) were positioned within the ACB channel with the benzylamide moiety oriented toward the membrane, and the isobutyl moiety pointing toward the catalytic triad, but differed for the position along the ACB channel, being **3** slightly shifted toward the catalytic triad. The more active compounds of the series, **11** and **15** showed high similarity to the binding mode predicted for **Ibu-AM5** (Supplementary Figure S2). All the compounds engaged one hydrogen bond (H-bond) interaction of the carbonyl with the Thr488 side chain, but differed for the positioning of the rest of the molecule (Figure 2). In particular, the isobutyl well matching the hydrophobic region formed by Ile491, Phe244, Ile238, and Leu192 in the case of **3**, or Phe381, Ala377, Phe432, and Leu380 in the case of amide **11**, and Leu192, Ile491, and Leu404 in the case of amide **15**. The binding mode adopted by amides **11** and **15** positioned the substituted benzyl ring in a similar position within the hydrophobic region at the gorge of MA channel and appeared favoured with respect to amide **3**, since they showed an additional H-bond interaction with Trp531 (amide **11**) or Asp403 (amide **15**).

With the aim to increase the hydrophilicity of the amides and to reduce the flexibility of the amide chain a new series of arylpiperazinoamides was designed. As indicated in Scheme 2, amides **18–27** were prepared by condensation between Ibuprofen (**1**) and aryl-substituted piperazines (**17**) using the EDC method. In general, all the arylpiperazinoamides displayed FAAH inhibitory activity better than benzylamide series. As shown in Table 2 the N-phenylpiperazinoamide **18** displayed inhibitory activity comparable to benzylamides **11** and **15**. The introduction of 4-methoxy group on the phenyl ring (amide **23**) did not change the activity, while the displacement of the methoxy group into the 3-position (amide **24**) led to a drop in activity. The replacement of 4-methoxy group with fluorine atom produced a reduction in activity (compound **22**, IC_50_ 17 μM). In contrast, the introduction in the same position of chlorine atom to give compound **20** improved ten-fold the inhibitory activity. Moving the chlorine from 4- to 3-position (amide **19**) did not affect the activity. However, the 3,4-dichlorophenyl substituted amide **21** that, contrary to expectation, showed inhibitory activity an order of magnitude lower potency than analogue **20**. The substitution of 3-chlorine of amide **19** with the methyl group to afford compound **25** caused a clear reduction in activity. The introduction of the second methyl group in 2-position (amide **26**) restored the activity, while the displacement of 3-methyl into 2-position produced loss of activity (amide **27**).

**Table 2.** Maximum percentage and IC50 values for inhibition of rat brain AEA hydrolysis by compounds **18–27**.

											
											

*The inhibition data was better fitted by a curve with a residual activity rather than a curve assuming 100% inhibition. The maximal inhibition is indicated (when it was greater than 50%), and the pI50 and IC50 values refer to the inhibitable portion of the curve. The inability of the compounds to produce a maximal inhibition was not investigated further.

**Figure 3.**
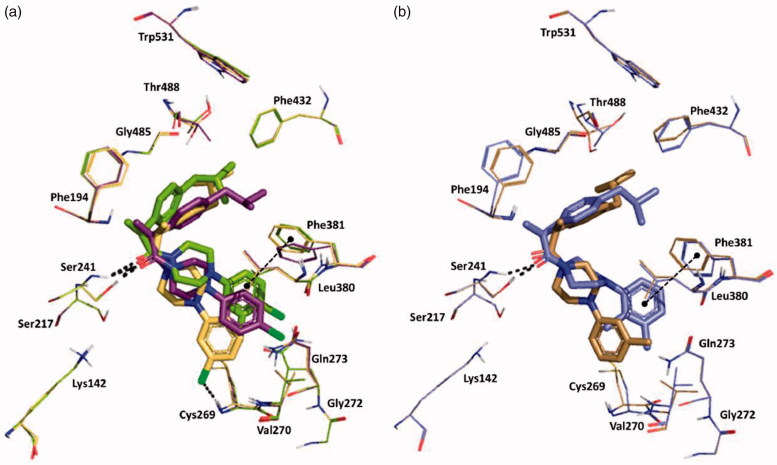
Superposition between the lowest emodel binding mode of (S)-piperazinoarylamides: (a) **19**, **20** and **21** depicted as yellow, purple and dark-green sticks, respectively; (b) **25** and **26** dysplayed as brown and violet sticks, respectively. rFAAH key residues involved in ligand interactions are displayed as lines coloured relatively as the interacting ligand. Hydrogen bond interactions and T-shaped π-π stacking interaction detected by Maestro 11.1 are shown as dashed black lines. Globally, the substitution of a chorine atom to the phenyl ring (compound **19**) allows an Hbond interaction with Cys269. The addition of a one more methyl group on the aromatic ring (amide **26**) does not affect the binding mode of the two ligands, except for the phenyl ring itself that in the case of amide **26** is slightly oriented toward the Phe381 residue engaging one T-shape π-π stacking interaction.

Induced-Fit docking at the enzyme active site revealed that all the arylpiperazinoamide derivatives adopted a different binding mode when compared to benzylamides or to the lead **Ibu-AM5**, binding deeper in the ACB, establishing, in most cases, direct interactions with at least one residue of the catalytic triad through the carbonyl of the amide, and the arylpiperazinoamide moiety entering the cytosolic port (CP). In order to clarify the SARs of this interesting novel series of ibuprofen derivatives, we analysed the binding mode of **19**, **20**, **21**, **25**, and **26**. Figure 3(a) represents the binding mode of amide **19**, showing two H-bonds with the catalytic triad residues Ser241 and Ser217, and a clear interaction of the chlorine with the NH of Cys269. Accordingly, the introduction of a second chlorine in 4-position, would clash with the side chain of Val270, thus forcing the 3,4-dichlorophenyl ring of compound **21** to move toward Phe381 (Figure 3(a)). This movement also induces a rearrangement of the isobutylphenyl moiety determining the disruption of the hydrophobic network that could account for the 10-fold loss of activity. Interestingly compound **20**, which showed the same FAAH inhibitory activity of analogue **19**, showed the same position as the isobutylphenyl moiety, albeit the 4-chlorophenyl ring assumed an intermediate conformation with respect to amides **19** and **21**. We also studied the binding of amides **25**, and **26** differing for the number and the position of methyl groups on the piperazine phenyl ring (Figure 3(b)). Docking results did not allow to give a clear explanation of how these subtle structural differences gave rise to the observed striking differences in activity. The two compounds indeed occupied the same binding pocket, and all established a H-bond with Ser241. Nevertheless, the introduction of the 2-methyl forced the phenyl ring to assume a perpendicular conformation with respect to the piperazine ring as an effect of steric hindrance with piperazine ring hydrogens. This led to different binding modes for 2-methylphenyl (compound **26**) and 3-methylphenyl analogue (**25**), that resulted in a different ability in engaging T-shape π-π stacking interactions with Phe381, only observed for 2-methyl derivative (Figure 3(b)). Albeit docking results gave interesting suggestions about some aspects of SAR of the benzyl- and the piperazine phenyl derivatives, the lack of correlation between IFDscores (Data not shown) and IC_50_ suggests some caution in interpreting the results.

As further modification, a methylene bridge was inserted between the piperazine and the aromatic ring. The amides **30–34** were prepared by condensation of ibuprofen (**1**) with N-BOC-piperazine (**29**), using the same EDC method described above. After BOC-deprotection, a benzyl group was added by reductive alkylation, treating the intermediate **6** with the suitable substituted arylaldehyde in presence of NaHCO_3_, NaBHAc_3_ in CH_2_Cl_2_ solution (Scheme 3). Unfortunately, this modification afforded the poor active analogues **30–34** (Table 3). This loss of activity could be attributed to the protonation of the nitrogen atom of the piperazine ring that is unfavourable in the hydrophobic environment of the FAAH channels. The induced fit docking of **30** and **32** confirmed this hypothesis, yielding binding modes characterised by the loss of interactions with Ser241, and by a closed conformation (Supplementary Figure S3), due largely to an intramolecular cation-π interaction, thus explaining the loss in the inhibition activity.

**Table 3.** Maximum percentage and IC50 values for inhibition of rat brain AEA hydrolysis by compounds **30–34**.

			

In conclusion, the present study has further explored the pharmacophore of **Ibu-AM5** with respect to its interaction with FAAH. Although the benzyl- and the piperazinoamides were logical areas to explore, neither set of derivatives improved upon the inhibitory potency of **Ibu-AM5**. Nevertheless, the arylpiperazinoamide derivatives showed a binding mode involving residues from the cytosolic port, which has been poorly explored as potential binding site of FAAH inhibitors and could, therefore, be considered interesting leads for the design of novel and more potent FAAH inhibitors.

## Supplementary Material

IENZ_1532418_Supplementary Material
