# Supplementary material for: Benzylamides and piperazinoarylamides of ibuprofen as fatty acid amide hydrolase inhibitors
Source: J Enzyme Inhib Med Chem. 2019 Jan 27;34(1):562–76. doi: 10.1080/14756366.2018.1532418 (PMC6352954; doi:10.1080/14756366.2018.1532418)
Supplement: IENZ_1532418_Supplementary Material [file IENZ_A_1532418_SM2700.pdf]

Supporting Informations

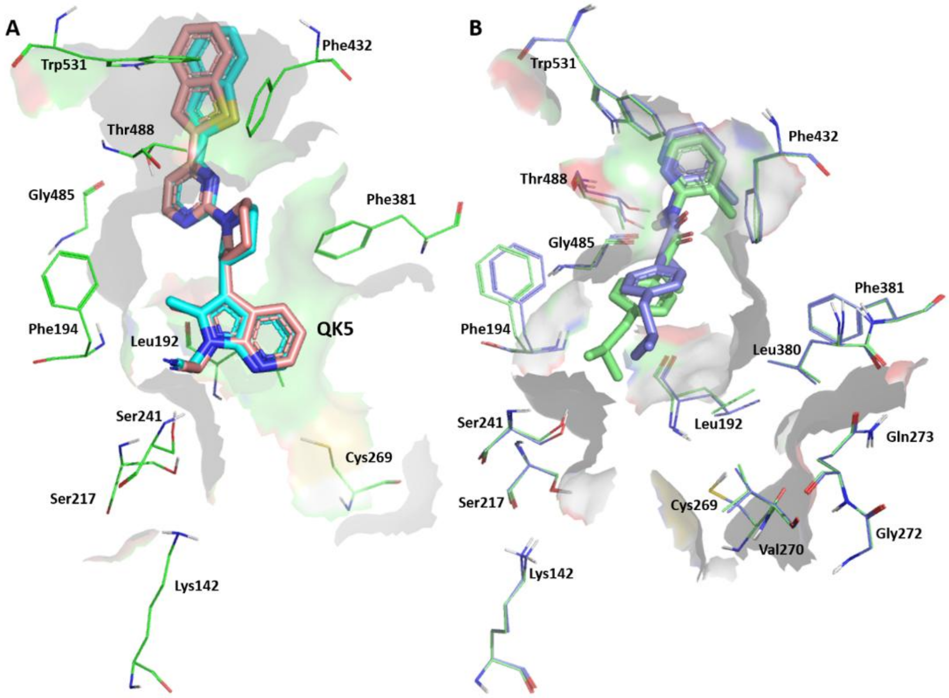

Figure S1. Superposition between: (**A**) The crystallographic pose of QK5 (cyan sticks) and the lowest IFDScore binding prediction (pink sticks) (RMSD: 0.58). (**B**) The binding mode of Ibu-AM5 found after MDs [1] (violet sticks) and the best IFDScore solution found by the IFD protocol (green sticks) (RMSD: 1.34).

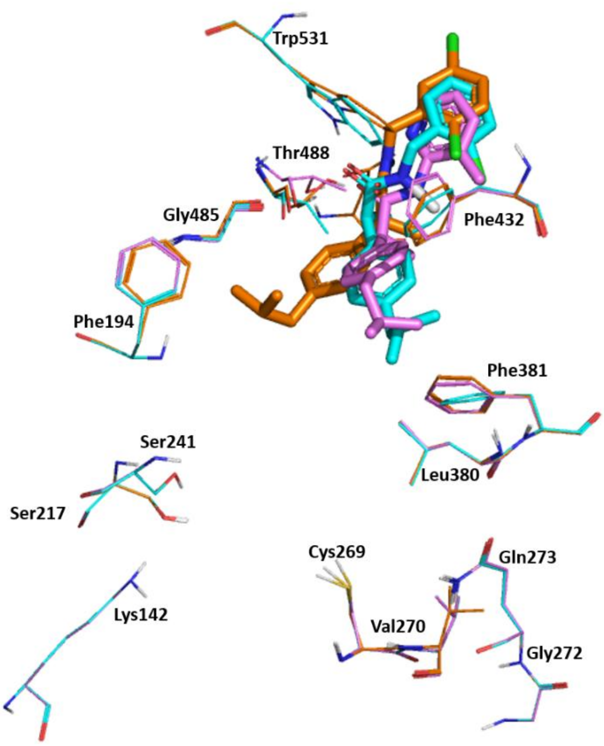

Figure S2. Superposition between the best emodel pose found for the (*S*)-enantiomers of benzylamides **11** and **15** with the binding mode of (*S*)-**Ibu-AM5**

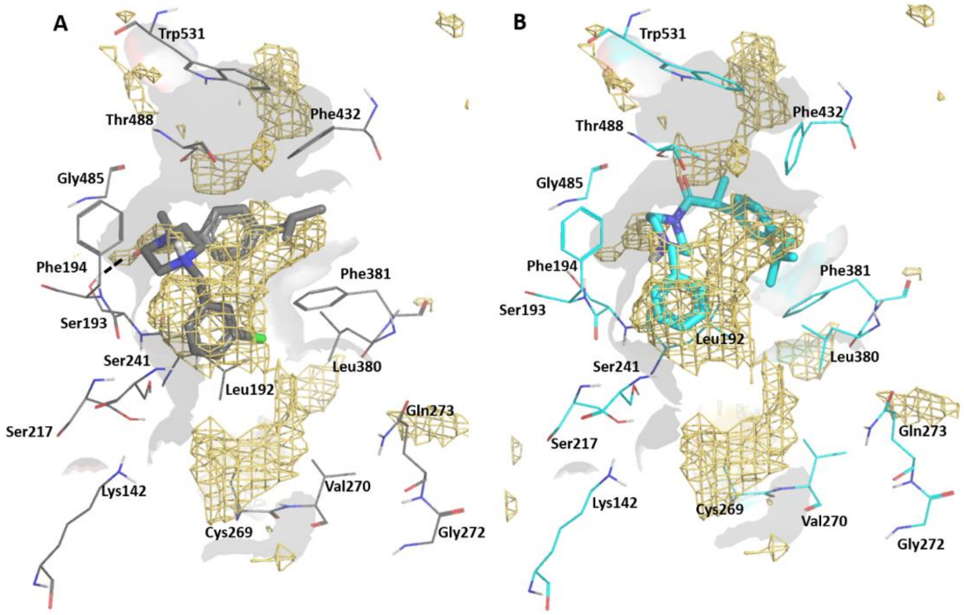

Figure S3: The lowest emodel binding mode of (*S*)-piperazinoarylamides (**A**) **32** and (**B**) **30**. The positively charged nitrogen atom on the piperazine ring, leads both ligands to assume a much-closed conformation, which hampers the inhibition activity.

IFD docking of (*R*)-enantiomers

Induced fit docking calculations were carried out on (*R*)-enantiomers of most active compounds of the benzylamide series (**11** and **15**) and of the piperazinoarylamide series (**19**, **20** and **26**). IFD calculations on the benzylamide derivatives (*R*)-**11** and (*R*)-**15** showed binding preferences different from those found for the corresponding (*S*)-enantiomers. The poses with the lowest Glide emodel energy showed the benzylamide moiety of both compounds lining the gorge of the MA channel, establishing hydrophobic interactions with Ile407, Met436 and Phe432 (Figure S4). The isobutyl moiety entered the ACB channel in two opposite directions, toward the catalytic triad in case of compound **15**, or toward the membrane in case of compound **11**. Both compounds also showed a H-bond with Thr488, with the NH group in case of compound **15** and with the carbonyl in case of **11**. Taken together IFD results on (*R*)-enantiomers of benzylamide series account for a binding mode interesting a different region with respect to (S)-enantiomers, therefore any hypothesis about stereoselectivity in FAAH interaction would be highly speculative.

IFD poses with the lowest glide emodel score of (*R*)-enantiomers of piperazinoarylamides occupied the same region of the ACB channel, but different in the orientation of the molecule. The two chlorine-substituted analogues (amides **19** and **20**) showed the carbonyl group H-bonded to Ser241 and the piperazinoaryl moiety entering the cytosolic port (CP), as previously described in the case of (*S*)-enantiomers. Interestingly, in case of compound **19,** albeit the poses for (*R*)- and (*S*)- enantiomers resulted superimposable, the different chirality induced a slight shift of 3-chlorophenyl ring determining the loss of the interaction of the chlorine with the NH of Cys269 (Figure S5A), that could suggest binding of (*R*)- **19** less favorable with respect to binding of (*S*)-**19**. On the other side IFD results on (*R*)- and (*S*)-**20** resulted very similar and didn't allowed to make any hypothesis about stereoselectivity in FAAH interaction .

IFD results on the (*R*)-2,3-dimethylphenyl derivative **26**, showed a binding orientation opposite to that found for (S) enantiomer (Figure S5C). The isobutyl moiety indeed entered the cytosolic port (CP) interacting with Val270 and Ile238, while the 2,3-dimethyl phenyl ring making hydrophobic contacts with Ile238, Leu380, Phe381 and Ile491. The different orientation induced a re-positioning of the carbonyl group resulting H-bonded to NH of Leu238. Overall, the best pose of (*R*)-**26** revealed a weakening of hydrophobic interactions established by the isobutyl moiety, and this could be in line with an expected reduction of activity with respect to (*S*)-**26.**

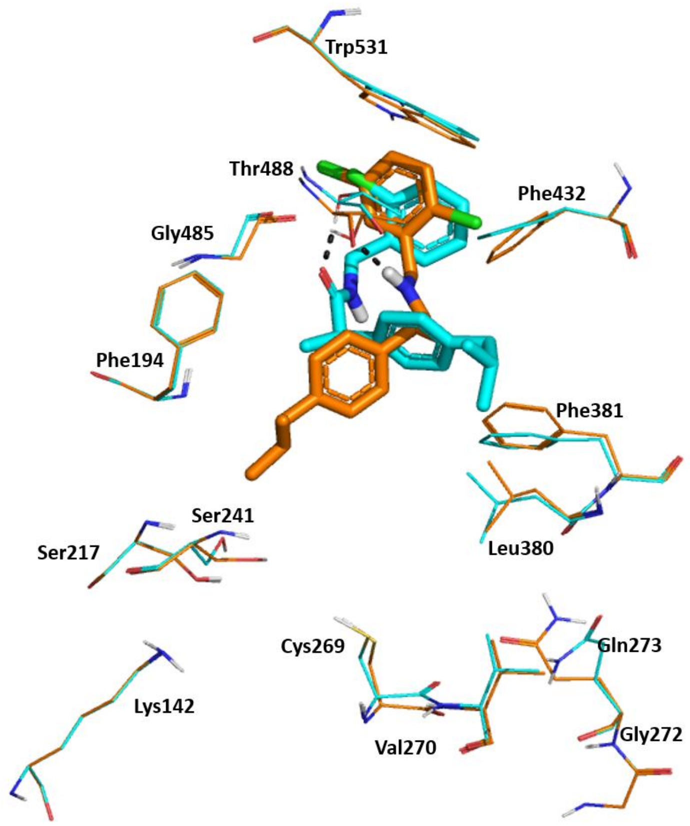

Figure S4: Superposition between the (*R*)-enantiomers of benzylamides compounds **11** (cyan stick) and **15** (orange stick). Hydrogen bond interactions are displayed as dashed black lines.

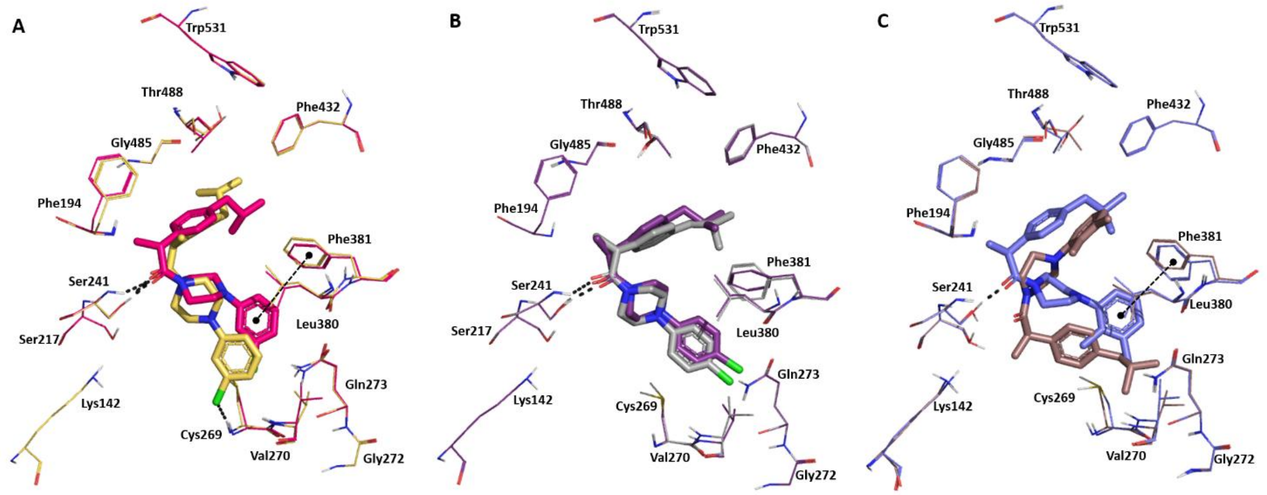

Figure S5: Superposition between the (*R*)- and (*S*)-enantiomers of the piperazinoarylamide series: (**A**) **19**; (**B**) **20**; (**C**) **26**. The (*R*)-enantiomers of **19**, **20** and **26** are shown as magenta, gray and brown sticks, respectively, while (*S*)-enantiomers of **19**, **20** and **26** are shown as yellow, purple and violet sticks, respectively. Hydrogen bond interactions are displayed as dashed black lines.

References

[1] Karlsson J.; Morgillo CM.; Deplano A.; Smaldone G.; Pedone E.; Luque F.J.; Svensson M.; Novellino E.; Congiu C.; Onnis V.; Catalanotti B.; Fowler C.J. Interaction of the N-(3-Methylpyridin-2-yl)amide Derivatives

of Flurbiprofen and Ibuprofen with FAAH: Enantiomeric Selectivity and Binding Mode. PLoS One., 2015, 10, e0142711.
